# A Multisource, Multilevel Contextual Database to Support Ending the HIV Epidemic Initiative: Protocol for Design, Construction, Harmonization, and Quality Assurance

**DOI:** 10.2196/100648

**Published:** 2026-11-03

**Authors:** Nikita Rao, Lingling Zheng, Wyatt Deihl, Raul U Hernandez-Ramirez, Donna Spiegelman, J Lucian Davis, Debbie Humphries

**Affiliations:** 1 Rigorous, Rapid and Relevant Evidence aDaptation and Implementation (R3EDI) for Ending the HIV Epidemic (EHE), Center for Methods in Implementation and Prevention Science (CMIPS), Yale School of Public Health, Yale University, PO Box 208034, New Haven, New Haven, CT, 06250, United States, 1 7707147619; 2 Partnerships and Development Department, CAN Community Health, Tampa, FL, United States; 3 Center for Interdisciplinary Research on AIDS (CIRA), Center for Methods in Implementation and Prevention Science (CMIPS), Yale School of Public Health, Yale University, New Haven, CT, United States; 4 Center for Methods in Implementation and Prevention Science (CMIPS), Yale School of Public Health, Yale University, New Haven, CT, United States; 5 Section of Pulmonary, Critical Care, and Sleep Medicine, Yale School of Medicine, Department of Epidemiology of Microbial Diseases, Yale School of Public Health, Yale University, New Haven, CT, United States

**Keywords:** HIV, implementation science, data science, exploration, data harmonization

## Abstract

**Background:**

The Ending the HIV Epidemic (EHE) initiative remains a national priority in the United States, aiming to reduce new HIV infections by 90% by 2030. As we cross the initiative’s midpoint, there is renewed commitment to strengthening the HIV workforce’s capacity to plan, implement, and sustain effective HIV prevention, treatment, and care interventions. Despite substantial improvement in HIV outcomes, uneven implementation of evidence-based interventions reflects persistent gaps between available evidence and its translation into locally actionable practice. Achieving EHE goals requires tailoring implementation to the diverse sociostructural conditions shaping HIV outcomes across jurisdictions. Research increasingly highlights the value of integrated, contextual data to strengthen public health decision-making. Linking indicators spanning multiple conceptual domains across various geographic levels can support a robust understanding of the distinct drivers of HIV outcomes, yet existing data systems remain fragmented. A harmonized, multisource, multilevel database is therefore essential to support targeted, needs-based, and data-driven implementation under the EHE initiative. We present a reproducible protocol detailing the transferable methodology of building and maintaining such a resource.

**Objective:**

This project aims to (1) build a high-quality contextual database integrating multiple data sources using transparent, replicable, and updatable methods and (2) develop and document systematic workflows for ongoing updates and quality assurance (QA) and support future use aligned with open-science frameworks and standard data practices.

**Methods:**

This project will follow best practices in data architecture, acquisition, standardization, and QA. For objective 1, we will integrate data across multiple geographic levels (eg, ZIP code, county) for 2020-2025, with measures categorized into conceptual domains (eg, epidemiologic and sociodemographic) guided by established theoretical frameworks. For objective 2, we will develop a tiered data structure using a GitHub repository to store documentation, processing scripts, and QA logs aligning with open science practices. Construction and QA methods were informed by targeted PubMed literature reviews. Data sources include existing data repositories, literature-identified datasets, and gray literature with consistent formatting and permissive terms of use suitable for web scraping. Stakeholder engagement will span all phases of database development, informing variable selection, usability, and validation to enable iterative refinement and revision.

**Results:**

Funding was obtained in August 2025. Literature reviews from October to November 2025 informed the database construction methods, source identification, and protocol development. Data acquisition, standardization, and QA began in May 2026, with acquisition remaining iterative. Cross-source integration, final QA checks, and dashboard development are expected to conclude in October 2026. Study results are anticipated for publication in summer 2027.

**Conclusions:**

This protocol will provide a reproducible, transferable model for building and maintaining a contextual data resource that could support public health planning and advance implementation science through more context-responsive decision-making under the EHE initiative.

## Introduction

The Ending the HIV Epidemic (EHE) initiative, coordinated by the US Department of Health and Human Services (HHS), is a national effort involving multiple federal, state, and local partners that aims to reduce new HIV infections by 90% by 2030 [1]. Although decades of biomedical and public health advances have improved HIV treatment, prevention, and care, uneven implementation of evidence-based interventions continues to hinder national progress toward EHE goals [2]. This challenge is evident in the stark concentration of HIV burden in a small number of counties and in the disproportionate impact on specific populations [3]. The challenge of translating evidence into action may intensify during periods of shifting policy and funding priorities, as the HIV workforce must make implementation decisions under increasingly constrained operational conditions [4]. Compounding these pressures, interruptions in the availability and continuity of data sources may affect researchers’ and practitioners’ ability to access the evidence needed to inform local implementation. A systematically assembled, well-documented database that tracks source-level changes can help buffer against these disruptions and support more resilient, evidence-informed decision-making.

A growing body of literature identifies the availability of diverse, multilevel data as a cornerstone of effective public health decision-making. In particular, contextual research evidence has emerged as essential for understanding the multifaceted nature of health systems and how they shape community health, as demonstrated by a recent systematic review examining data use in government-led public health interventions [5]. This is further supported by evidence emphasizing the role of contextual factors in influencing health outcomes. For example, recent county-level analyses using composite measures estimate that socioeconomic factors alone account for nearly half of county-level variation in health outcomes, far exceeding the contribution of clinical care [6]. A systematic review similarly found that socioeconomic, cultural, and environmental contexts shape the effectiveness of public health interventions yet remain underreported in the current evidence base [7]. National surveillance data further document substantial disparities in social determinants of health (SDOH) across populations, affirming the importance of contextual factors in understanding risk exposure and health status [8]. SDOH represent one key domain within a broader contextual framework that also encompasses environmental, structural, and geographic influences on health. Throughout this protocol, we use the term contextual indicators to refer to measurable, area-level characteristics of the environments in which people live that shape their health outcomes, spanning epidemiologic, sociodemographic, economic, structural, and policy-related conditions across geographic levels. The need for integrated, multilevel evidence is further illustrated by syndemic processes, through which shared social and structural determinants simultaneously shape multiple epidemics. Alongside intersectionality frameworks, this perspective also highlights how overlapping systems of inequality shape both exposure to risk and access to care [9,10]. For example, the opioid crisis has reshaped HIV transmission dynamics by exploiting overlapping social vulnerabilities [11]. Because SDOH operate across multiple levels, with some rooted in individual circumstances and others clustering geographically at the community level, individual-level data are often both unavailable and insufficient for capturing these contextual nuances. Addressing these complex drivers of health would therefore be aided by a data infrastructure that aggregates, standardizes, and links existing publicly available data across systems and scales, improving accessibility of multilevel contextual information to support public health intervention planning and practice. The development of such an infrastructure, however, requires confronting persistent challenges in how data systems are currently built and integrated.

Advances in contextual database design show that integrating data across multiple levels is necessary to capture the macro-micro linkages through which broader contextual factors shape individual and population health trends [12,13]. Yet, existing data systems remain disconnected, developed for differing purposes, varying scales, and disparate knowledge domains [12], contributing to a siloed understanding of contextual factors in practice. Data harmonization therefore plays a key role in generating evidence that is comparable, analytically robust, and relevant for policy. A multisource, multilevel database could substantially advance targeted implementation efforts under the EHE initiative.

As part of a broader effort to enhance the rigor and impact of HIV implementation research across EHE priority jurisdictions [14], the primary objective of this project is to build a reproducible, high-quality, contextual database, constituting the second version of an existing database currently housed on the Yale Center for Methods in Implementation and Prevention Science (CMIPS) website [15]. This database is distinguished from existing contextual data resources by its integration and harmonization of multiple sources into a single, HIV-focused, multilevel resource. While AIDSVu primarily visualizes HIV surveillance data alongside selected social determinants, County Health Rankings & Roadmaps ranks counties on broad health measures, and US Census data provide raw counts and individual demographic measurements, each is typically organized at a single geographic level and for a distinct purpose. In contrast, the present database links harmonized contextual indicators across multiple geographic levels and conceptual domains, tailored specifically to EHE priority jurisdictions, and documents source-level provenance and updates to support transparent, reproducible reuse.

A secondary objective is to create transparent and systematic workflows for ongoing updates, quality assurance (QA), and future analyses, thereby providing resources for generating reproducible and transparent knowledge to support EHE-aligned implementation research. In this protocol, we describe the systematic processes and phases guiding database development, articulate the anticipated value of the linked dataset, and discuss anticipated barriers and facilitators, along with plans for dissemination and future analytic work.

We present this work as a reproducible contextual data infrastructure protocol with transparent methodology for assembling, harmonizing, documenting, and maintaining a multilevel contextual data resource. Its central contribution is not the resulting dataset alone but the integrated and reproducible process of building and sustaining such a resource spanning data architecture, harmonization, QA, documentation, and stakeholder engagement.

## Methods

We will use a structured, theory-informed approach to develop the contextual HIV database, using conceptual frameworks to guide domain organization, identifying relevant data sources through a targeted and pragmatic literature review, and applying standardized procedures for database architecture, data acquisition, harmonization, QA, and stakeholder engagement. Together, these components will support the development of a rigorous, reproducible, and scalable data resource, as described below.

### Conceptual Framework

In this database, measures will be organized into conceptual domains informed by Ecological Systems Theory (EST) and the First Nations Perspective on Health and Wellness, 2 complementary frameworks that together address distinct aspects of how context shapes HIV outcomes (Figure 1). Rather than offering redundant accounts of the same phenomena, each framework answers a different organizing question: EST specifies *at what* levels contextual indicators operate, while the First Nations Perspective informs *what is counted as health-relevant* and whose perspective defines it. First proposed by Urie Bronfenbrenner [16], EST frames lifespan development as occurring within nested systems of influence, from immediate family environments to larger societal structures [17]. This nested structure maps directly onto the multilevel architecture of our database, in which contextual indicators are assembled across individual, neighborhood, county, state, and national scales, allowing the framework to organize variables according to the level at which each indicator operates. EST is well-suited to HIV research, where outcomes are shaped by interactions between individual-level influences and macrolevel factors [18,19].

The First Nations perspective complements EST by supplying a holistic and inherently collective concept of health, one that emerges from the interplay of social, cultural, and relational systems rather than residing solely within the individual [20,21]. This perspective broadens the contextual domains we capture to include community and relational conditions that many health-systems models tend to omit, but that are increasingly recognized as central to HIV prevention and care in the United States. Although this perspective originates in an Indigenous health context, its emphasis on collective and structural determinants is transferable to US HIV implementation settings, where disparities are similarly patterned by community-level and structural conditions rather than individual behavior alone.

Together, these frameworks provide a theoretically grounded structure for organizing contextual factors into conceptual domains, illustrating how data across multiple levels interact to influence health outcomes. Figure 1 presents this structure as a proximal-to-distal organization of domains, ranging from more immediate, individual-level determinants to broader structural and systemic influences. This arrangement is not ranked but simply shows domains in relation to one another, consistent with the manuscript’s emphasis on interconnected, multilevel influences. Each conceptual domain was drawn from these frameworks and then translated into specific database variables. For example, the economic stability domain maps to indicators such as housing stability status, median household income, and median hours worked. These domains were reviewed with stakeholders, including implementation scientists, people with lived experience of HIV, a research librarian, and a potential end user of the database, who examined the planned variables captured within these domains and how well they relate to one another. This review led to refinements, such as the addition of policy-related indicators in the social and community context domain, including reentry program funding per capita, sex work criminalization and enforcement intensity, and drug paraphernalia laws. In the context of HIV research, this approach supports the development of comprehensive, culturally relevant implementation strategies that address layered vulnerabilities in HIV prevention and care.

**Figure 1.**
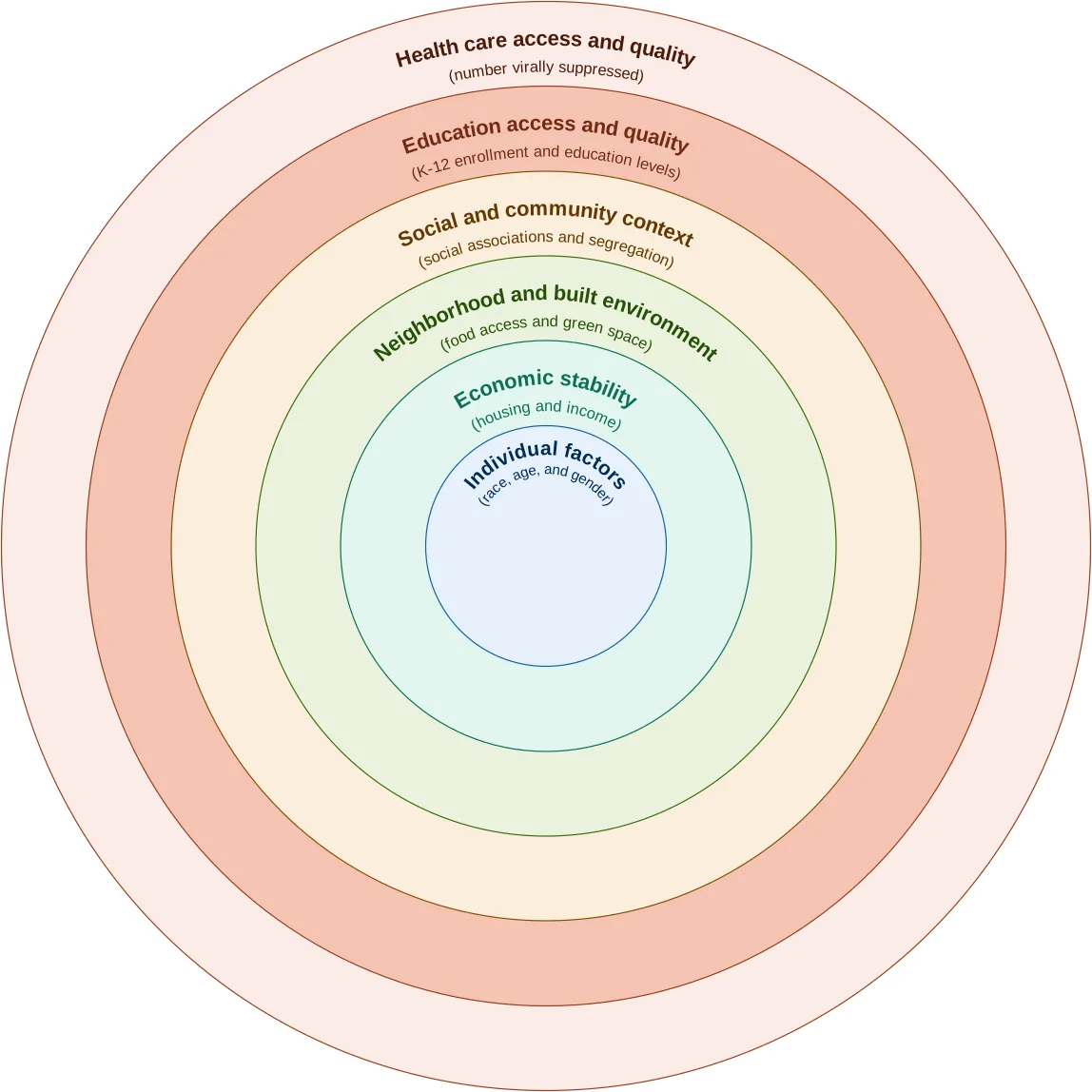
The relational HIV ecosystem model.

### Literature Search

To inform database construction, harmonization, and QA procedures and to ensure alignment with best practices in community engagement [22], we conducted 2 targeted, pragmatic literature reviews. Rather than aiming to provide an exhaustive synthesis of the literature, these reviews were intended to identify usable methods and candidate contextual variables sufficient to inform our build. We therefore prioritized relevance and feasibility over exhaustive retrieval. Working with implementation researchers and a librarian from the Yale School of Public Health, we refined search strategies for (1) a methodological literature search focused on database design and management approaches and (2) a separate literature search identifying proxy variables, defined as indicators used to approximate relevant contextual conditions that are not directly measured, to ensure that contextual indicators are captured. These 2 reviews served complementary purposes: the methodological review identified approaches for building and managing the database (data management, harmonization, linkage, QA, and web-scraping), while the proxy-variable review identified its contextual content (candidate variables and data sources). In consultation with the librarian, we focused on PubMed as the primary database, for its broad indexing of health and biomedical literature. Recognizing that methodological literature in fields such as data engineering, information technology, and geospatial science is indexed across additional platforms, we supplemented the searches by informally reviewing citations and references from included studies and by conducting complementary gray-literature searches. Searching was considered sufficient once it yielded methods and variables adequate to inform the database design. To document this process transparently, we provide full search strings, filters, dates, and result counts in Multimedia Appendix 1 and screening criteria in Multimedia Appendix 2.

We imported search results into their respective libraries using Zotero citation management software (version 7.0; Corporation for Digital Scholarship) [23] and subsequently into Rayyan [24], a free online screening tool for literature reviews. Based on predefined inclusion and exclusion criteria (Multimedia Appendix 2), one researcher (NR) reviewed the methodological literature search results and identified approaches related to data management, web-scraping, data integration, and data linkage. Because this review addressed a narrow question under strict eligibility criteria, 7 studies ultimately met the criteria and informed the framework, reflecting the specificity of the question and the review’s pragmatic scope rather than a search limitation. A second researcher (LZ) conducted the proxy variable literature search, which further helped identify traditional datasets (eg, American Community Survey [ACS], AIDSVu [Rollins School of Public Health, Emory University, in partnership with Gilead Sciences, Inc, and the Center for AIDS Research at Emory University]) for inclusion in our database. Complementary gray literature searches were also conducted to identify unstructured, web-based sources appropriate for web scraping. Screening for both reviews was conducted by a single reviewer, without independent dual screening or interrater reliability assessment, consistent with the targeted pragmatic design of this literature search. While this may introduce selection bias, its impact on the database build is expected to be limited.

### Overview of Database Structure and Architecture

Database development will follow a structured approach guided by best practices in data architecture, acquisition, standardization, and QA to create a multisource, multiyear, and multilevel contextual resource to support HIV implementation research. We will integrate national-, state-, county-, and ZIP Code-level data for the years 2020‐2025, accounting for the differing update cycles of contributing sources, including annual, biennial, and 5-year releases (eg, ACS 5-y estimates). All measures will be categorized into clearly defined conceptual domains informed by EST and the First Nations Perspective on Health and Wellness to facilitate future analyses.

We will use a 4-tier data architecture to ensure transparent and reproducible data management. Tier 1 will consist of raw and unmodified source files alongside their original documentation. In Tier 2, datasets will be cleaned and standardized by applying naming conventions, recoding data structures, aligning geographical identifiers, and tagging years. Tier 3 will integrate all standardized sources into a harmonized master file using predefined merging and conflict-resolution rules [25]. QA procedures will be applied across Tiers 2 and 3 to assess data completeness, consistency, and integration. Tier 4 will provide the final dataset, comprising curated variables, derived measures, and conceptually grouped indicators to support future analyses. The compiled database is structured as a single integrated, long-format file rather than a multitable relational database. Each row represents one county in a year, uniquely identified by its Federal Information Processing Standards (FIPS) code and year. Standardized identifier columns (FIPS code, state, county name, and year) appear first in every version of the file, followed by all substantive variables, each prefixed by the source (eg, acs_ and aidsvu_) to prevent naming conflicts. This structure is built in two stages: (1) datasets from the same source are first stacked longitudinally across years and (2) these datasets are then integrated across sources by joining on the shared county FIPS identifier. All versions of the integrated file are saved under version-controlled naming conventions to support reproducibility. This 4-tier system provides a stable foundation for ongoing maintenance and future scalability, reflecting standard data design and management frameworks [22,26]. To maintain version control and ensure transparency and traceability throughout all stages of database construction, we will implement a complementary documentation and version-control plan. During the prebuild phase, all project materials will be housed in a shared folder for centralized access. Excel logs will be used to maintain detailed records of search strategies, inclusion/exclusion decisions, and candidate database summaries. Once the database build is initiated, we will migrate all documentation and code to a shared GitHub repository with version control and issue tracking to align with open-science standards and support long-term reproducibility. In addition to GitHub-based version control of code and documentation, each public release of the harmonized dataset will be maintained as a fixed, versioned snapshot hosted on Yale-managed infrastructure, accompanied by documentation describing changes relative to the prior release. Prior versions will remain accessible so that external users can retrieve and cite the specific version of the database used in a given analysis, even as the database continues to be updated.

To document variables and sources, we will maintain a structured data dictionary aligned with the Dublin Core Metadata Element Set [27], capturing each source’s title, origin, publisher, date, coverage, license, and description. Source-level provenance, including the originating dataset, access date, and source version or release, will be recorded in the dictionary and updated as sources are refreshed, providing a timestamped history on GitHub.

For each derived variable, the data dictionary will record its source variables, the transformation applied, and a reference to the script that generates it. The transformation code will be kept under version control in GitHub. The data dictionary will show how each variable is produced, while GitHub’s commit history and issue tracking will provide a time-stamped record of source-level changes, derivations, and updates over time. Together, these practices align with current FAIR (Findability, Accessibility, Interoperability, and Reusability) data principles that guide our approach (Multimedia Appendix 3) [28].

### Data Acquisition, Collection, and Harmonization

The final selection of datasets and web-based sources to be included in the contextual database will be derived from three sources: (1) data sources identified in the existing CMIPS database, (2) datasets identified in the proxy-variable literature search, and (3) reports and gray-literature sources with consistent formatting and permissive terms of use suitable for web scraping.

Consistent with the goal of building a comprehensive contextual database, our approach prioritizes inclusion rather than selective exclusion. Candidate sources will be retained unless they lack a permissive license or a format amenable to reliable extraction. To refine and validate this preliminary list, we will share it with stakeholders, including the AIDSVu development team and potential end users, who will review it for relevance and utility. Stakeholder input will serve in an advisory role to identify additional sources for consideration to expand and prioritize candidate data sources rather than to remove them, keeping in line with the database’s comprehensive intent. Suggested sources will be incorporated where they meet licensing requirements; if a source does not meet these requirements, this exclusion reason will be documented.

Because the database is intended to be inclusive, sources are retained even when they overlap or conflict. Rather than resolving cases by exclusion, we preserve the sources and document overlaps, discrepancies, and source information in the data dictionary and methods documentation, so that end users can determine usage for their own purposes. Similarly, incomplete data will be retained rather than discarded and flagged and described in the data dictionary and methods documentation alongside missing, suppressed, and otherwise limited values. All inclusion decisions, data sources, and data quality descriptions will be documented in a log, accompanied by justification notes to maintain transparency and reproducibility.

Full datasets will be manually downloaded directly from the source when available. If unavailable, semiautomated web-scraping methods adapted from the web scraping, text mining, and spatial overlay method [29] will be applied to extract raw data values from unstructured sources such as PDFs and web pages. Web scraping tasks will be performed using the rvest package for static data [30] or the RSelenium package [31] for dynamic webpages. This approach supports reproducible and transparent collection of nontabular data, ensuring broader coverage of relevant contextual indicators.

Individual dataset cleaning and harmonization will include applying standardized naming conventions, addressing missing and inconsistent values, converting measures into uniform units and scales, and using data crosswalks to map and align geographical elements (eg, FIPS codes, Core Based Statistical Areas, ZIP codes, etc). Geographic units that require translation to the county level, including sources that do not report FIPS codes directly, will be mapped using the US Department of Housing and Urban Development United States Postal Service (USPS) ZIP-County Crosswalk File to derive a standardized FIPS code prior to matching. We will link datasets at the level of EHE jurisdictions (US counties, states, and equivalent areas). Matching will be performed on the FIPS code, as it is a standardized geographic identifier common to all our sources following crosswalk translation. Because FIPS codes are exact, standardized identifiers, this deterministic approach yields distinct matches and avoids the ambiguity of name-based matching, and conflicting matches are not anticipated. Jurisdiction names will be used only as a secondary check if they differ across sources and will be manually reviewed to identify and correct any formatting or naming discrepancies, based on the recommendation of Cao et al [25]. For example, a county in both AIDSVu and ACS is matched on its 5-digit FIPS code, with its name checked for formatting differences. Sources with differing update frequencies are accommodated through the matching process. Annually updated sources are matched by both FIPS code and year, while sources that update less frequently (eg, ACS 5-y estimates) are matched by FIPS code alone and populate only the years corresponding to their actual data release; years without a new source update are left blank rather than carrying forward the prior value, so that users are not at risk of interpreting a repeated value as an independently observed annual measurement. Users conducting analyses that require complete year-by-year coverage are responsible for determining and applying their own approach to these blank years (eg, carrying values forward, interpolation, or restricting analysis to observed years), consistent with the assumptions appropriate to their specific research question. This will be noted in the data dictionary and data methods for each nonannual indicator, along with the source’s typical update frequency, so users can identify affected variables before conducting longitudinal analyses. We cross-referenced the US Census Bureau’s official record of substantial county boundary changes [32] against the 57 EHE priority jurisdictions and confirmed no overlap during the 2020‐2025 study period (eg, Connecticut’s 2022 transition to planning regions and county-equivalent boundary adjustments in Alaska and Virginia all fall outside EHE-designated counties and states), supporting the stability of FIPS-based geographic identifiers for the scope of this database.

### QA

Our QA approach will follow the Bray and Parkin [26] framework, which assesses data quality in terms of completeness, comparability, timeliness, and validity across all data sources. In accordance with methods outlined by Palis et al [33], we will assess these benchmarks in Tiers 2 through 4 of database construction. Systematic reabstraction (including re-extracting key variables from original data sources) and re-derivation (independently reproducing derived variables and indicators) audits will be conducted manually on a stratified sample (eg, by HIV burden, region, and rurality). Audit scope, including variable selection and the sample size for each cycle, is determined based on the dataset under review. As a general guidance, audits sample at least 5% of records per stratum, with a minimum of 10 records per stratum, and are conducted by an auditor independent of the person who originally processed the data. A target of ≥95% agreement is consistent with reabstraction reliability standards reported for large national health registries such as the National Cancer Database [33] and with Landis and Koch [34]’s widely used interpretation of κ≥0.8 as indicating almost perfect agreement. When agreement or κ targets are not met, findings are escalated for team review to determine whether the discrepancy reflects a systematic processing error requiring correction and reprocessing of downstream steps, an isolated error requiring targeted correction, or an ambiguity in documented procedures requiring classification before reprocessing. These results will be summarized in a report for team discussion and action [35], in which each finding is classified as resolved, requiring corrective action with an assigned responsible party and target date, or accepted as a known limitation with the rationale documented. Insights from Zhang et al [36] will further inform our QA structure, including governance and data-security considerations relevant for future integration of sensitive or clinic-level data. Database updates will follow documented and version-controlled workflows encompassing data acquisition and harmonization alongside reapplication of QA checks to ensure consistency, transparency, reproducibility, and scalability over time. The update cadence will be source-specific, aligning with each source’s release schedule rather than a fixed timeline. Where feasible, automated update pipelines will be implemented for publicly available data on predictable schedules, while other sources will undergo manual review and updates to preserve data integrity.

### Stakeholder Engagement

We will engage stakeholders during all phases of contextual database development, following principles from implementation science and registry-development frameworks [22,33,37,38]. Stakeholders were identified through purposive sampling, drawing on known experts and prospective users relevant to each phase of development; individuals were approached as their specific expertise or perspective became pertinent to the work at hand. During the planning and literature-search phases, the team engaged local implementation science researchers and a research librarian to refine the conceptual domains, search strategy, and inclusion criteria. Additional stakeholder involvement is planned throughout subsequent phases of the workflow, as illustrated in Figure 2.

**Figure 2.**
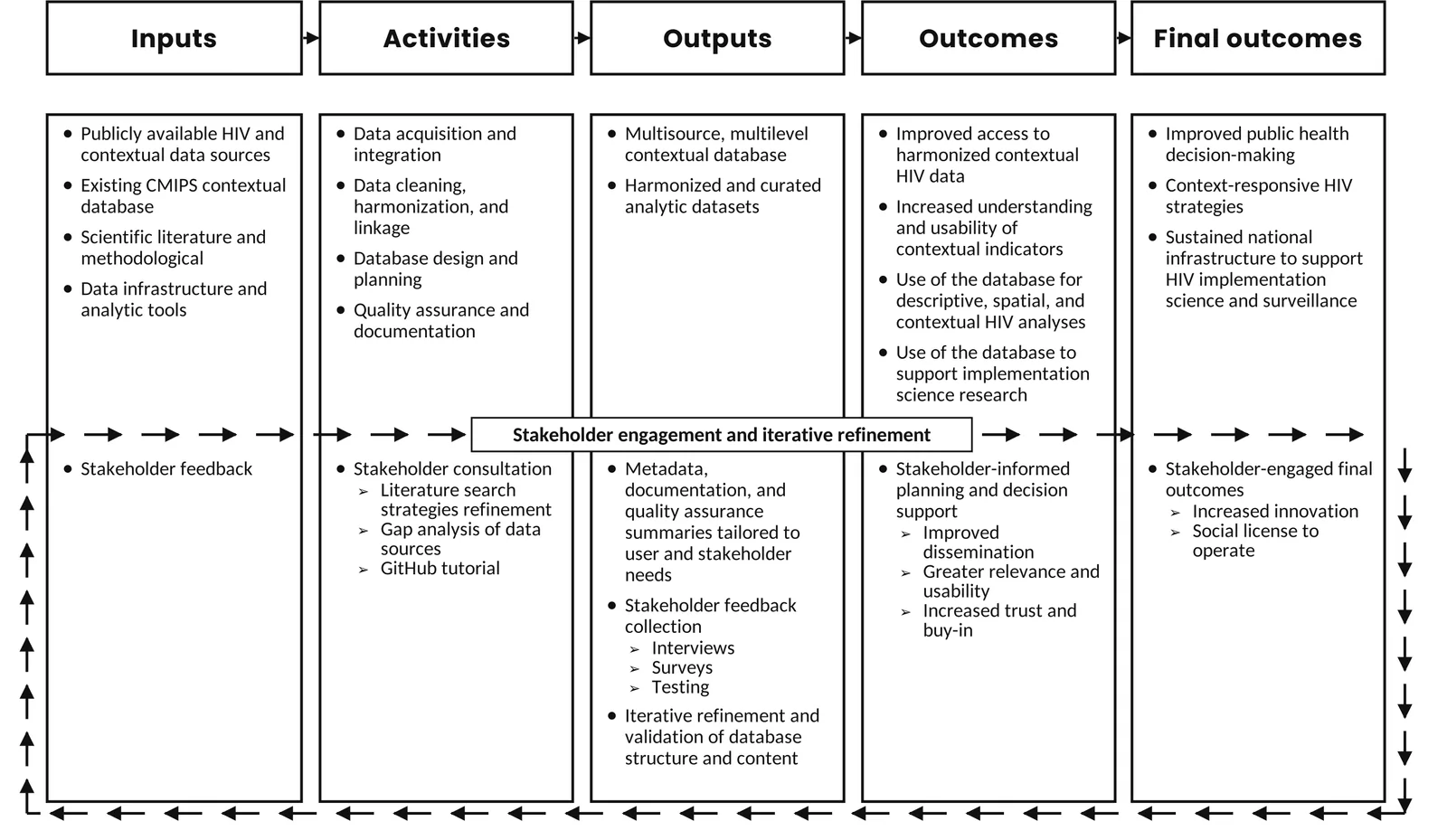
Logic model for contextual database development, use, and stakeholder engagement. CMIPS: Center for Methods in Implementation and Prevention Science.

This figure presents a logic model linking project inputs, activities, outputs, outcomes, and anticipated final outcomes, with stakeholder engagement and iterative refinement integrated across all stages of database development. This includes engagement with data scientists, people living with HIV, and prospective end users such as implementation science researchers and other public health professionals. Consultations will be conducted iteratively rather than on a fixed schedule, tied to development milestones shown in Figure 2, with specific stakeholders engaged as relevant to each stage. For example, we consulted an expert in web-scraping and version control (GitHub) to inform our data-engineering approach and a prospective end user to identify additional contextual variables of practical value. Stakeholder feedback will inform iterative revisions to the identification of data sources, the data architecture, QA procedures, user interface features, and dissemination strategies. We will develop a stakeholder engagement matrix mapping key advisory groups, including clinicians, non-governmental organization workers, policymakers, researchers, and people living with HIV, to ensure comprehensive coverage. Stakeholders serve in an advisory capacity and provide input and recommendations, while final decisions regarding database design, content, and implementation rest with the research team. No stakeholder holds a formal governance, approval, or oversight role. Because stakeholders are engaged for distinct areas of expertise, their recommendations typically address different aspects of the project and rarely conflict; where recommendations do diverge, the research team will reconcile them considering the database’s goals of comprehensiveness, feasibility, and user relevance and will document the rationale. Outreach is already underway, with structured consultations planned at key development milestones to ensure that the database is relevant, rigorous, responsive to user needs, and practice for real-world implementation.

### Ethical Considerations

This study uses publicly available data aggregated at the ZIP code or higher geographic level and does not include individual-level or identifiable information. Data sources originate from federal, academic, state, or local public health agencies. Stakeholder engagement activities were intended to inform database development; no personal data were collected in these consultations, and this is not formal human subjects research. This study therefore qualifies as nonhuman subjects research under US federal regulations [39]. Institutional review is not required and therefore was not sought.

All data use will comply with applicable terms of use, licensing, and attribution requirements for each source. Should future expansions incorporate sensitive or restricted-access datasets, appropriate data-sharing agreements, governance structures, and institutional approvals will be pursued.

## Results

This project was funded in August 2025. Our data-method search, combining the initial and supplemental searches described below, yielded 80 unique PubMed articles after duplicate removal. During the first-stage title and abstract screening, 16 studies were selected for full-text review, of which 7 met inclusion criteria following second-stage screening. Studies were excluded if they were reports or planned registries using data within clinical trials (Figure S1 in Multimedia Appendix 4). The proxy-variable search yielded 273 unique PubMed articles after duplicate removal. Of these, 63 studies were advanced to full-text review based on title and abstract screening, and 38 met inclusion criteria after second-stage screening. Studies were excluded if they were nonquantitative, duplicative, or relied on internal measures (Figure S2 in Multimedia Appendix 4).

As the initial data-methods search produced only 5 eligible studies, we conducted a supplemental structured literature search focused on database development methods to broaden our understanding of best practices and enhance methodological rigor. This supplemental search identified 2 additional studies, bringing the combined total reported above to 7. Detailed search strategies for all searches are provided in Multimedia Appendix 1.

Identification of the initial datasets for inclusion in the contextual database was finalized in April 2026. Data acquisition, standardization, and QA began in May 2026 and are ongoing, yielding to date 130 county-level datasets from 9 sources and 150 state-level datasets from 8 sources (Multimedia Appendix 5), with acquisition remaining iterative. Cross-source data harmonization, final QA checks, and dashboard development are expected to conclude in October 2026, with development of a public-facing domain including database access and additional tools, planned for a December 2026 release. Study results are anticipated for publication in summer 2027.

## Discussion

This protocol outlines the planned development of a contextual database intended to support future research, implementation science, and public health decision-making under the EHE initiative. Beyond database construction, this work has potential implications for analytical applications, practical utility, and dissemination. These considerations collectively highlight the potential value of the database as a scalable and reproducible resource for contextual HIV research and implementation planning.

### Analytic Implications

Our contextual database is designed to facilitate a wide range of analytic applications examining how multilevel social, structural, and demographic factors shape HIV outcomes, service availability, and burden across the United States. By integrating and harmonizing multisource and multilevel contextual indicators across SDOH domains, we anticipate that the database will provide a foundation for future analyses that inform epidemiological research, implementation strategies, and policy-relevant decision-making. It may be particularly useful for community-based organizations and other practitioners responsible for implementing EHE priorities, for whom analytic capacity and access to integrated contextual data may be limited. By reducing the burden of identifying, sourcing, and linking relevant datasets, the database may also support a more holistic understanding of SDOH by bringing together epidemiologic, demographic, and structural indicators that are often analyzed separately. The analytic applications described below are illustrative of the database’s intended uses. The feasibility and validity of any specific analysis will depend on the completeness, temporal coverage, geographic consistency, and outcome availability of the underlying data, which vary across indicators and jurisdictions.

First, the database is intended to support descriptive and analytic epidemiological analyses, including examining spatial patterns in relation to contextual indicators, identifying clusters of high HIV burden associated with structural and community-level determinants, and characterizing temporal trends in HIV outcomes. Beyond description, analytic epidemiologic applications may include identifying determinants of HIV incidence and service usage, quantifying the contribution of structural and contextual factors to geographic variation in outcomes, and, where data completeness and longitudinal coverage permit, supporting causal inference approaches such as difference-in-differences or interrupted time series analyses where longitudinal data permit. These analyses may inform surveillance and help to set priorities for the EHE initiative and related efforts.

Second, the database may be relevant for implementation science applications, particularly those related to HIV epidemiology and service delivery. Contextual indicators spanning structural, health systems, and neighborhood domains could be used to evaluate moderators of prevention and treatment interventions, such as structural barriers, service availability, policy environments, and socioeconomic conditions. Additionally, they may help identify geographic areas with high HIV burden, unmet needs, or service gaps. Subject to data availability, these data may enable the estimation of the incremental risk associated with specific epidemiologic and service delivery indicators across diverse contexts, providing a quantitative basis for directing resources, tailoring interventions, and informing policy. These applications align with the National Institutes of Health (NIH) priorities to end the HIV epidemic through implementation research on medical and behavioral interventions [40].

Finally, the database may support public health planning by enabling advanced analytic techniques, potentially including predictive and simulation modeling. For instance, machine learning prediction models and prioritization models could support resource allocation by accounting for higher-level health determinants, such as structural and community conditions [41], provided that data of sufficient quality, completeness, and temporal depth are available. Such applications would require careful validation before informing practice. We will also launch a public-facing website featuring interactive dashboards to help users visualize cross-domain contextual indicators, thereby supporting stakeholders in decision-making for EHE implementation. In doing so, the database aims to strengthen research and dissemination by creating reproducible, generalizable knowledge to advance EHE goals [14].

### Dissemination

Dissemination of this work will occur through multiple channels, including peer-reviewed manuscripts describing the database architecture, methods, and analyses; presentations at scientific conferences and stakeholder meetings; a public-facing dashboard to support EHE-related planning and decision-making; public GitHub repositories containing analytic code, data-processing scripts, QA logs, and metadata; and comprehensive technical documentation and user guides.

Dedicated workshops and consultation sessions will also be conducted to facilitate technical proficiency in database navigation for people living with HIV, community organization staff, and the broader scientific community.

### Strengths and Limitations

This project has several important strengths related to both database development and its anticipated utility. This database provides a comprehensive integration of multiyear, multilevel HIV and contextual indicators, assembled through a systematic search and data collection process. The reproducible workflow supports transparency through version control with GitHub, structured Excel logs, and metadata tracking. Robust QA procedures follow the Bray and Parkin [26] framework and are in accordance with established guidelines. Stakeholder engagement throughout all phases of database development enhances relevance, usability, and alignment with EHE priorities. The tiered architecture allows for scalability, enabling expansion to additional years, indicators, or geographies as new data become available, while supporting a range of analytic applications.

Several project-level limitations should be noted. Because the database relies on publicly available secondary data, source availability, update schedules, and completeness may vary across indicators and over time; these are tracked through systematic QA procedures, with findings recorded in the data dictionary and metadata to inform end users. Variability in data quality and completeness may require complex harmonization procedures; missing value patterns, functionally unusable data, and notable discontinuities in indicator values are systematically assessed during data cleaning and QA checks, with findings documented in the data dictionary to support transparency and informed analytic decision-making. Gaps in geographic coverage or changes in reporting practices may limit comparability across geographies, and transparent documentation of source-level characteristics is provided to help users interpret these differences accordingly. Public data constraints, such as suppressed values, may limit analytic precision and the ability to capture all implementation-relevant local contexts; these are similarly flagged in the data dictionary to inform analytic decisions. The current database focuses on EHE jurisdictions, which may limit generalizability; however, the tiered architecture is designed to support expansion to additional geographies as needs and resources allow. As with all analyses relying on aggregate data, findings reflect population-level patterns and do not capture individual-level heterogeneity, and results should be interpreted at the aggregate level.

### Conclusions

As the EHE initiative crosses its midpoint, strengthening the capacity of the HIV workforce to make precise, context-sensitive decisions at the local level has become increasingly critical. Central to this effort is the development of tools to bridge the gap between available evidence and locally relevant implementation strategies. The central contribution of this work is a reproducible contextual data infrastructure protocol rather than the resulting dataset in isolation. This includes an integrated, transparent, and transferable methodology for assembling, harmonizing, documenting, and maintaining a multilevel contextual data resource. The HIV contextual database will provide a comprehensive, reproducible, and scalable resource to support multilevel public health research, implementation science, and strategic planning. By integrating multiyear and multilevel contextual indicators through systematic, transparent, and reproducible methods, the database will enhance the capacity to examine the multidimensional factors shaping HIV outcomes and burdens across the United States.

The tiered architecture, standardized procedures for obtaining, cleaning, and harmonizing data, and consistent QA checks ensure that the database can be maintained and expanded as new data become available and as emerging contextual measures become relevant. This platform aims to improve understanding of the structural and social determinants of HIV, support evidence-based decision-making, and contribute to efforts to reduce the HIV burden and advance health outcomes within the EHE initiative.

## Supplementary material

10.2196/100648 Multimedia Appendix 1 Search strategies.

10.2196/100648 Multimedia Appendix 2 Screening criteria for literature search.

10.2196/100648 Multimedia Appendix 3 Implementation of FAIR data principles.

10.2196/100648 Multimedia Appendix 4 PRISMA 2020 flow diagrams for literature searches.

10.2196/100648 Multimedia Appendix 5 Data sources included in the database to date.

## Abbreviations

ACS: American Community Survey
CMIPS: Center for Methods in Implementation and Prevention Science
EHE: Ending the HIV Epidemic
EST: Ecological Systems Theory
FAIR: Findability, Accessibility, Interoperability, and Reusability
FIPS: Federal Information Processing Standards
HHS: United States Department of Health and Human Services
NIH: National Institutes of Health
PRISMA: Preferred Reporting Items for Systematic Reviews and Meta-Analyses
QA: quality assurance
SDOH: social determinants of health
USPS: United States Postal Service
