# Supplementary material for: A Multisource, Multilevel Contextual Database to Support Ending the HIV Epidemic Initiative: Protocol for Design, Construction, Harmonization, and Quality Assurance
Source: JMIR Res Protoc. 2026 Nov 3;15:e100648. doi: 10.2196/100648 (PMC13730519; doi:10.2196/100648)
Supplement: Supplementary file 1 [file resprot-v15-e100648-s001.docx]

**Multimedia Appendix 1**

Search Strategies

**PubMed Proxy Variable Literature Search**

Filters used on Search Number 10 and over: in the last 5 years, English, Humans

| **Search Number** | **Query** | **Search Details** | **Results** |
| --- | --- | --- | --- |
| 22 | ((HIV [tiab]) AND ((y_5[Filter]) AND (humans[Filter]) AND (english[Filter]))) AND (((((hiv[MeSH Terms]) AND (socioeconomic factors[MeSH Terms])) OR (social determinants of health[MeSH Terms])) OR (( income [Title/Abstract] OR "education*" [Title/Abstract] OR "wealth*"[Title/Abstract] OR "occupation*"[Title/Abstract] OR "poverty"[Title/Abstract] OR "asset ind*"[Title/Abstract] OR "inde*"[Title/Abstract]) AND ("indicator*"[Title/Abstract] OR "measure*"[Title/Abstract] OR "prox*"[Title/Abstract])) AND ((y_5[Filter]) AND (humans[Filter]) AND (english[Filter]))) AND (united states[MeSH Terms] AND ((y_5[Filter]) AND (humans[Filter]) AND (english[Filter]))) AND ((y_5[Filter]) AND (humans[Filter]) AND (english[Filter]))) | ("HIV"[Title/Abstract] AND ("2020/10/09 00:00":"3000/01/01 05:00"[Date - Publication] AND "humans"[MeSH Terms] AND "english"[Language]) AND ((("HIV"[MeSH Terms] AND "socioeconomic factors"[MeSH Terms]) OR "social determinants of health"[MeSH Terms] OR (("income"[Title/Abstract] OR "education*"[Title/Abstract] OR "wealth*"[Title/Abstract] OR "occupation*"[Title/Abstract] OR "poverty"[Title/Abstract] OR "asset ind*"[Title/Abstract] OR "inde*"[Title/Abstract]) AND ("indicator*"[Title/Abstract] OR "measure*"[Title/Abstract] OR "prox*"[Title/Abstract]))) AND ("2020/10/09 00:00":"3000/01/01 05:00"[Date - Publication] AND "humans"[MeSH Terms] AND "english"[Language]) AND ("united states"[MeSH Terms] AND ("2020/10/09 00:00":"3000/01/01 05:00"[Date - Publication] AND "humans"[MeSH Terms] AND "english"[Language])) AND ("2020/10/09 00:00":"3000/01/01 05:00"[Date - Publication] AND "humans"[MeSH Terms] AND "english"[Language]))) AND ((y_5[Filter]) AND (humans[Filter]) AND (english[Filter])) | 274 |
| 21 | ((((hiv[MeSH Terms]) AND (socioeconomic factors[MeSH Terms])) OR (social determinants of health[MeSH Terms])) OR (( income [Title/Abstract] OR "education*" [Title/Abstract] OR "wealth*"[Title/Abstract] OR "occupation*"[Title/Abstract] OR "poverty"[Title/Abstract] OR "asset ind*"[Title/Abstract] OR "inde*"[Title/Abstract]) AND ("indicator*"[Title/Abstract] OR "measure*"[Title/Abstract] OR "prox*"[Title/Abstract])) AND ((y_5[Filter]) AND (humans[Filter]) AND (english[Filter]))) AND (united states[MeSH Terms] AND ((y_5[Filter]) AND (humans[Filter]) AND (english[Filter]))) | ((("hiv"[MeSH Terms] AND "socioeconomic factors"[MeSH Terms]) OR "social determinants of health"[MeSH Terms] OR (("income"[Title/Abstract] OR "education*"[Title/Abstract] OR "wealth*"[Title/Abstract] OR "occupation*"[Title/Abstract] OR "poverty"[Title/Abstract] OR "asset ind*"[Title/Abstract] OR "inde*"[Title/Abstract]) AND ("indicator*"[Title/Abstract] OR "measure*"[Title/Abstract] OR "prox*"[Title/Abstract]))) AND ("2020/10/09 00:00":"3000/01/01 05:00"[Date - Publication] AND "humans"[MeSH Terms] AND "english"[Language]) AND ("united states"[MeSH Terms] AND ("2020/10/09 00:00":"3000/01/01 05:00"[Date - Publication] AND "humans"[MeSH Terms] AND "english"[Language]))) AND ((y_5[Filter]) AND (humans[Filter]) AND (english[Filter])) | 13,306 |
| 20 | united states[MeSH Terms] | ("united states"[MeSH Terms]) AND ((y_5[Filter]) AND (humans[Filter]) AND (english[Filter])) | 144,540 |
| 12 | (((hiv[MeSH Terms]) AND (socioeconomic factors[MeSH Terms])) OR (social determinants of health[MeSH Terms])) OR (( income [Title/Abstract] OR "education*" [Title/Abstract] OR "wealth*"[Title/Abstract] OR "occupation*"[Title/Abstract] OR "poverty"[Title/Abstract] OR "asset ind*"[Title/Abstract] OR "inde*"[Title/Abstract]) AND ("indicator*"[Title/Abstract] OR "measure*"[Title/Abstract] OR "prox*"[Title/Abstract])) | (("hiv"[MeSH Terms] AND "socioeconomic factors"[MeSH Terms]) OR "social determinants of health"[MeSH Terms] OR (("income"[Title/Abstract] OR "education*"[Title/Abstract] OR "wealth*"[Title/Abstract] OR "occupation*"[Title/Abstract] OR "poverty"[Title/Abstract] OR "asset ind*"[Title/Abstract] OR "inde*"[Title/Abstract]) AND ("indicator*"[Title/Abstract] OR "measure*"[Title/Abstract] OR "prox*"[Title/Abstract]))) AND ((y_5[Filter]) AND (humans[Filter]) AND (english[Filter])) | 210,547 |
| 19 | (HIV [tiab]) AND (socioeconomic factors[MeSH Terms]) OR (social determinants of health[MeSH Terms]) OR (( income [Title/Abstract] OR "education*" [Title/Abstract] OR "wealth*"[Title/Abstract] OR "occupation*"[Title/Abstract] OR "poverty"[Title/Abstract] OR "asset ind*"[Title/Abstract] OR "inde*"[Title/Abstract]) AND ("indicator*"[Title/Abstract] OR "measure*"[Title/Abstract] OR "prox*"[Title/Abstract])) AND ((y_5[Filter]) AND (humans[Filter]) AND (english[Filter])) | ((("HIV"[Title/Abstract] AND "socioeconomic factors"[MeSH Terms]) OR "social determinants of health"[MeSH Terms] OR (("income"[Title/Abstract] OR "education*"[Title/Abstract] OR "wealth*"[Title/Abstract] OR "occupation*"[Title/Abstract] OR "poverty"[Title/Abstract] OR "asset ind*"[Title/Abstract] OR "inde*"[Title/Abstract]) AND ("indicator*"[Title/Abstract] OR "measure*"[Title/Abstract] OR "prox*"[Title/Abstract]))) AND ("2020/10/09 00:00":"3000/01/01 05:00"[Date - Publication] AND "humans"[MeSH Terms] AND "english"[Language])) AND ((y_5[Filter]) AND (humans[Filter]) AND (english[Filter])) | 211,356 |
| 18 | ((HIV [tiab]) AND (socioeconomic factors[MeSH Terms]) OR (social determinants of health[MeSH Terms]) OR (( income [Title/Abstract] OR "education*" [Title/Abstract] OR "wealth*"[Title/Abstract] OR "occupation*"[Title/Abstract] OR "poverty"[Title/Abstract] OR "asset ind*"[Title/Abstract] OR "inde*"[Title/Abstract]) AND ("indicator*"[Title/Abstract] OR "measure*"[Title/Abstract] OR "prox*"[Title/Abstract])) | (("HIV"[Title/Abstract] AND "socioeconomic factors"[MeSH Terms]) OR "social determinants of health"[MeSH Terms] OR (("income"[Title/Abstract] OR "education*"[Title/Abstract] OR "wealth*"[Title/Abstract] OR "occupation*"[Title/Abstract] OR "poverty"[Title/Abstract] OR "asset ind*"[Title/Abstract] OR "inde*"[Title/Abstract]) AND ("indicator*"[Title/Abstract] OR "measure*"[Title/Abstract] OR "prox*"[Title/Abstract]))) AND ((y_5[Filter]) AND (humans[Filter]) AND (english[Filter])) | 211,356 |
| 17 | ((HIV [tiab]) AND ((y_5[Filter]) AND (humans[Filter]) AND (english[Filter]))) AND (socioeconomic factors[MeSH Terms]) OR (social determinants of health[MeSH Terms]) OR (( income [Title/Abstract] OR "education*" [Title/Abstract] OR "wealth*"[Title/Abstract] OR "occupation*"[Title/Abstract] OR "poverty"[Title/Abstract] OR "asset ind*"[Title/Abstract] OR "inde*"[Title/Abstract]) AND ("indicator*"[Title/Abstract] OR "measure*"[Title/Abstract] OR "prox*"[Title/Abstract])) | (("HIV"[Title/Abstract] AND ("2020/10/09 00:00":"3000/01/01 05:00"[Date - Publication] AND "humans"[MeSH Terms] AND "english"[Language]) AND "socioeconomic factors"[MeSH Terms]) OR "social determinants of health"[MeSH Terms] OR (("income"[Title/Abstract] OR "education*"[Title/Abstract] OR "wealth*"[Title/Abstract] OR "occupation*"[Title/Abstract] OR "poverty"[Title/Abstract] OR "asset ind*"[Title/Abstract] OR "inde*"[Title/Abstract]) AND ("indicator*"[Title/Abstract] OR "measure*"[Title/Abstract] OR "prox*"[Title/Abstract]))) AND ((y_5[Filter]) AND (humans[Filter]) AND (english[Filter])) | 211,356 |
| 14 | (HIV [tiab]) | ("HIV"[Title/Abstract]) AND ((y_5[Filter]) AND (humans[Filter]) AND (english[Filter])) | 48,132 |
| 13 | (HIV) | ("hiv"[MeSH Terms] OR "hiv"[All Fields]) AND ((y_5[Filter]) AND (humans[Filter]) AND (english[Filter])) | 54,557 |
| 11 | (((hiv[MeSH Terms]) AND (socioeconomic factors[MeSH Terms])) OR (social determinants of health[MeSH Terms])) OR (( income [Title/Abstract] OR "education*" [Title/Abstract] OR "wealth*"[Title/Abstract] OR "occupation*"[Title/Abstract] OR "poverty"[Title/Abstract] OR "asset ind*"[Title/Abstract] OR "inde*"[Title/Abstract]) AND ("indicator*"[Title/Abstract] OR "measure*"[Title/Abstract] OR "prox*"[Title/Abstract])) | (("hiv"[MeSH Terms] AND "socioeconomic factors"[MeSH Terms]) OR "social determinants of health"[MeSH Terms] OR (("income"[Title/Abstract] OR "education*"[Title/Abstract] OR "wealth*"[Title/Abstract] OR "occupation*"[Title/Abstract] OR "poverty"[Title/Abstract] OR "asset ind*"[Title/Abstract] OR "inde*"[Title/Abstract]) AND ("indicator*"[Title/Abstract] OR "measure*"[Title/Abstract] OR "prox*"[Title/Abstract]))) AND ((y_5[Filter]) AND (english[Filter])) | 328,686 |
| 10 | (((hiv[MeSH Terms]) AND (socioeconomic factors[MeSH Terms])) OR (social determinants of health[MeSH Terms])) OR (( income [Title/Abstract] OR "education*" [Title/Abstract] OR "wealth*"[Title/Abstract] OR "occupation*"[Title/Abstract] OR "poverty"[Title/Abstract] OR "asset ind*"[Title/Abstract] OR "inde*"[Title/Abstract]) AND ("indicator*"[Title/Abstract] OR "measure*"[Title/Abstract] OR "prox*"[Title/Abstract])) | (("hiv"[MeSH Terms] AND "socioeconomic factors"[MeSH Terms]) OR "social determinants of health"[MeSH Terms] OR (("income"[Title/Abstract] OR "education*"[Title/Abstract] OR "wealth*"[Title/Abstract] OR "occupation*"[Title/Abstract] OR "poverty"[Title/Abstract] OR "asset ind*"[Title/Abstract] OR "inde*"[Title/Abstract]) AND ("indicator*"[Title/Abstract] OR "measure*"[Title/Abstract] OR "prox*"[Title/Abstract]))) AND (y_5[Filter]) | 334,333 |
| 5 | ( income [Title/Abstract] OR "education*" [Title/Abstract] OR "wealth*"[Title/Abstract] OR "occupation*"[Title/Abstract] OR "poverty"[Title/Abstract] OR "asset ind*"[Title/Abstract] OR "inde*"[Title/Abstract]) AND ("indicator*"[Title/Abstract] OR "measure*"[Title/Abstract] OR "prox*"[Title/Abstract]) | ("income"[Title/Abstract] OR "education*"[Title/Abstract] OR "wealth*"[Title/Abstract] OR "occupation*"[Title/Abstract] OR "poverty"[Title/Abstract] OR "asset ind*"[Title/Abstract] OR "inde*"[Title/Abstract]) AND ("indicator*"[Title/Abstract] OR "measure*"[Title/Abstract] OR "prox*"[Title/Abstract]) | 1,034,436 |
| 3 | social determinants of health[MeSH Terms] | "social determinants of health"[MeSH Terms] | 9,393 |
| 2 | socioeconomic factors[MeSH Terms] | "socioeconomic factors"[MeSH Terms] | 541,332 |
| 1 | hiv[MeSH Terms] | "hiv"[MeSH Terms] | 111,220 |

**Methods Literature Search 1**

Filters used on all Search Numbers: in the last 5 years, English, Humans

| **Search Number** | **Query** | **Search Details** | **Results** |
| --- | --- | --- | --- |
| 9 | ((protocol[ti] OR methodology[ti] OR framework[ti] OR "study protocol"[ti] AND ((y_5[Filter]) AND (humans[Filter]) AND (english[Filter]))) AND (database[ti] OR "data registry"[ti] or registry[ti] AND ((y_5[Filter]) AND (humans[Filter]) AND (english[Filter])))) AND ("web scrap*"[tw] OR "data scrap*"[tw] OR "data collection"[tw] OR "web mining"[tw] OR "data extraction" [tw] OR "data harvest*"[tw] AND ((y_5[Filter]) AND (humans[Filter]) AND (english[Filter]))) | (("protocol"[Title] OR "methodology"[Title] OR "framework"[Title] OR "study protocol"[Title]) AND ("2020/10/09 00:00":"3000/01/01 05:00"[Date - Publication] AND "humans"[MeSH Terms] AND "english"[Language]) AND (("database"[Title] OR "data registry"[Title] OR "registry"[Title]) AND ("2020/10/09 00:00":"3000/01/01 05:00"[Date - Publication] AND "humans"[MeSH Terms] AND "english"[Language])) AND (("web scrap*"[Text Word] OR "data scrap*"[Text Word] OR "data collection"[Text Word] OR "web mining"[Text Word] OR "data extraction"[Text Word] OR "data harvest*"[Text Word]) AND ("2020/10/09 00:00":"3000/01/01 05:00"[Date - Publication] AND "humans"[MeSH Terms] AND "english"[Language]))) AND ((y_5[Filter]) AND (humans[Filter]) AND (english[Filter])) | 56 |
| 8 | ((protocol[ti] OR methodology[ti] OR framework[ti] OR "study protocol"[ti] or construction AND ((y_5[Filter]) AND (humans[Filter]) AND (english[Filter])) AND ((y_5[Filter]) AND (humans[Filter]) AND (english[Filter]))) AND (database[ti] AND ((y_5[Filter]) AND (humans[Filter]) AND (english[Filter])))) AND ("web scrap*"[tw] OR "data scrap*"[tw] OR "data collection"[tw] OR "web mining"[tw] OR "data extraction" [tw] OR "data harvest*"[tw] AND ((y_5[Filter]) AND (humans[Filter]) AND (english[Filter]))) | (("protocol"[Title] OR "methodology"[Title] OR "framework"[Title] OR "study protocol"[Title] OR ("construct s"[All Fields] OR "constructed"[All Fields] OR "constructing"[All Fields] OR "construction"[All Fields] OR "constructions"[All Fields] OR "constructive"[All Fields] OR "constructively"[All Fields] OR "constructs"[All Fields] OR "dna recombinant"[Supplementary Concept] OR "dna recombinant"[All Fields] OR "construct"[All Fields] OR "dna, recombinant"[MeSH Terms] OR ("dna"[All Fields] AND "recombinant"[All Fields]) OR "recombinant dna"[All Fields])) AND ("2020/10/09 00:00":"3000/01/01 05:00"[Date - Publication] AND "humans"[MeSH Terms] AND "english"[Language]) AND ("2020/10/09 00:00":"3000/01/01 05:00"[Date - Publication] AND "humans"[MeSH Terms] AND "english"[Language]) AND ("database"[Title] AND ("2020/10/09 00:00":"3000/01/01 05:00"[Date - Publication] AND "humans"[MeSH Terms] AND "english"[Language])) AND (("web scrap*"[Text Word] OR "data scrap*"[Text Word] OR "data collection"[Text Word] OR "web mining"[Text Word] OR "data extraction"[Text Word] OR "data harvest*"[Text Word]) AND ("2020/10/09 00:00":"3000/01/01 05:00"[Date - Publication] AND "humans"[MeSH Terms] AND "english"[Language]))) AND ((y_5[Filter]) AND (humans[Filter]) AND (english[Filter])) | 16 |
| 7 | ((protocol[ti] OR methodology[ti] OR framework[ti] OR "study protocol"[ti] AND ((y_5[Filter]) AND (humans[Filter]) AND (english[Filter]))) AND (database[ti] AND ((y_5[Filter]) AND (humans[Filter]) AND (english[Filter])))) AND ("web scrap*"[tw] OR "data scrap*"[tw] OR "data collection"[tw] OR "web mining"[tw] OR "data extraction" [tw] OR "data harvest*"[tw] AND ((y_5[Filter]) AND (humans[Filter]) AND (english[Filter]))) | (("protocol"[Title] OR "methodology"[Title] OR "framework"[Title] OR "study protocol"[Title]) AND ("2020/10/09 00:00":"3000/01/01 05:00"[Date - Publication] AND "humans"[MeSH Terms] AND "english"[Language]) AND ("database"[Title] AND ("2020/10/09 00:00":"3000/01/01 05:00"[Date - Publication] AND "humans"[MeSH Terms] AND "english"[Language])) AND (("web scrap*"[Text Word] OR "data scrap*"[Text Word] OR "data collection"[Text Word] OR "web mining"[Text Word] OR "data extraction"[Text Word] OR "data harvest*"[Text Word]) AND ("2020/10/09 00:00":"3000/01/01 05:00"[Date - Publication] AND "humans"[MeSH Terms] AND "english"[Language]))) AND ((y_5[Filter]) AND (humans[Filter]) AND (english[Filter])) | 9 |
| 6 | database[ti] | ("database"[Title]) AND ((y_5[Filter]) AND (humans[Filter]) AND (english[Filter])) | 11,174 |
| 5 | ((protocol[ti] OR methodology[ti] OR framework[ti] OR "study protocol"[ti] or construction AND ((y_5[Filter]) AND (humans[Filter]) AND (english[Filter])) AND ((y_5[Filter]) AND (humans[Filter]) AND (english[Filter]))) AND ("web scrap*"[tw] OR "data scrap*"[tw] OR "data collection"[tw] OR "web mining"[tw] OR "data extraction" [tw] OR "data harvest*"[tw] AND ((y_5[Filter]) AND (humans[Filter]) AND (english[Filter])))) AND (database[ti] OR "data registry"[ti] or registry[ti] AND ((y_5[Filter]) AND (humans[Filter]) AND (english[Filter]))) | (("protocol"[Title] OR "methodology"[Title] OR "framework"[Title] OR "study protocol"[Title] OR ("construct s"[All Fields] OR "constructed"[All Fields] OR "constructing"[All Fields] OR "construction"[All Fields] OR "constructions"[All Fields] OR "constructive"[All Fields] OR "constructively"[All Fields] OR "constructs"[All Fields] OR "dna recombinant"[Supplementary Concept] OR "dna recombinant"[All Fields] OR "construct"[All Fields] OR "dna, recombinant"[MeSH Terms] OR ("dna"[All Fields] AND "recombinant"[All Fields]) OR "recombinant dna"[All Fields])) AND ("2020/10/09 00:00":"3000/01/01 05:00"[Date - Publication] AND "humans"[MeSH Terms] AND "english"[Language]) AND ("2020/10/09 00:00":"3000/01/01 05:00"[Date - Publication] AND "humans"[MeSH Terms] AND "english"[Language]) AND (("web scrap*"[Text Word] OR "data scrap*"[Text Word] OR "data collection"[Text Word] OR "web mining"[Text Word] OR "data extraction"[Text Word] OR "data harvest*"[Text Word]) AND ("2020/10/09 00:00":"3000/01/01 05:00"[Date - Publication] AND "humans"[MeSH Terms] AND "english"[Language])) AND (("database"[Title] OR "data registry"[Title] OR "registry"[Title]) AND ("2020/10/09 00:00":"3000/01/01 05:00"[Date - Publication] AND "humans"[MeSH Terms] AND "english"[Language]))) AND ((y_5[Filter]) AND (humans[Filter]) AND (english[Filter])) | 68 |
| 4 | protocol[ti] OR methodology[ti] OR framework[ti] OR "study protocol"[ti] or construction AND ((y_5[Filter]) AND (humans[Filter]) AND (english[Filter])) | (("protocol"[Title] OR "methodology"[Title] OR "framework"[Title] OR "study protocol"[Title] OR ("construct s"[All Fields] OR "constructed"[All Fields] OR "constructing"[All Fields] OR "construction"[All Fields] OR "constructions"[All Fields] OR "constructive"[All Fields] OR "constructively"[All Fields] OR "constructs"[All Fields] OR "dna recombinant"[Supplementary Concept] OR "dna recombinant"[All Fields] OR "construct"[All Fields] OR "dna, recombinant"[MeSH Terms] OR ("dna"[All Fields] AND "recombinant"[All Fields]) OR "recombinant dna"[All Fields])) AND ("2020/10/09 00:00":"3000/01/01 05:00"[Date - Publication] AND "humans"[MeSH Terms] AND "english"[Language])) AND ((y_5[Filter]) AND (humans[Filter]) AND (english[Filter])) | 173,427 |
| 3 | "web scrap*"[tw] OR "data scrap*"[tw] OR "data collection"[tw] OR "web mining"[tw] OR "data extraction" [tw] OR "data harvest*"[tw] | ("web scrap*"[Text Word] OR "data scrap*"[Text Word] OR "data collection"[Text Word] OR "web mining"[Text Word] OR "data extraction"[Text Word] OR "data harvest*"[Text Word]) AND ((y_5[Filter]) AND (humans[Filter]) AND (english[Filter])) | 47,859 |
| 2 | database[ti] OR "data registry"[ti] or registry[ti] | ("database"[Title] OR "data registry"[Title] OR "registry"[Title]) AND ((y_5[Filter]) AND (humans[Filter]) AND (english[Filter])) | 23,357 |
| 1 | protocol[ti] OR methodology[ti] OR framework[ti] OR "study protocol"[ti] | ("protocol"[Title] OR "methodology"[Title] OR "framework"[Title] OR "study protocol"[Title]) AND ((y_5[Filter]) AND (humans[Filter]) AND (english[Filter])) | 48,516 |

**Methods Literature Search 2**

Filters used on all Search Numbers: in the last 5 years, English, Humans

| **Search Number** | **Query** | **Search Details** | **Results** |
| --- | --- | --- | --- |
| 5 | (((("web scraping" OR "web-mined" OR "crowdsourced") AND ((y_5[Filter]) AND (humans[Filter]) AND (english[Filter]))) AND ((health OR public health) AND ((y_5[Filter]) AND (humans[Filter]) AND (english[Filter])))) AND ((spatial OR GIS OR "spatial overlay" OR geocoding) AND ((y_5[Filter]) AND (humans[Filter]) AND (english[Filter])))) AND ((tutorial OR framework OR method*) AND ((y_5[Filter]) AND (humans[Filter]) AND (english[Filter]))) | (("web scraping"[All Fields] OR "web-mined"[All Fields] OR "crowdsourced"[All Fields]) AND ("2020/10/16 00:00":"3000/01/01 05:00"[Date - Publication] AND "humans"[MeSH Terms] AND "english"[Language]) AND (("health"[MeSH Terms] OR "health"[All Fields] OR "health s"[All Fields] OR "healthful"[All Fields] OR "healthfulness"[All Fields] OR "healths"[All Fields] OR ("public health"[MeSH Terms] OR ("public"[All Fields] AND "health"[All Fields]) OR "public health"[All Fields])) AND ("2020/10/16 00:00":"3000/01/01 05:00"[Date - Publication] AND "humans"[MeSH Terms] AND "english"[Language])) AND (("spatial"[All Fields] OR "spatialization"[All Fields] OR "spatializations"[All Fields] OR "spatialized"[All Fields] OR "spatially"[All Fields] OR ("proc acm sigspatial int conf adv inf"[Journal] OR "gis"[All Fields]) OR "spatial overlay"[All Fields] OR ("geocode"[All Fields] OR "geocoded"[All Fields] OR "geocoder"[All Fields] OR "geocodes"[All Fields] OR "geographic mapping"[MeSH Terms] OR ("geographic"[All Fields] AND "mapping"[All Fields]) OR "geographic mapping"[All Fields] OR "geocoding"[All Fields])) AND ("2020/10/16 00:00":"3000/01/01 05:00"[Date - Publication] AND "humans"[MeSH Terms] AND "english"[Language])) AND (("tutorial"[All Fields] OR "tutorials"[All Fields] OR ("framework"[All Fields] OR "framework s"[All Fields] OR "frameworks"[All Fields]) OR "method*"[All Fields]) AND ("2020/10/16 00:00":"3000/01/01 05:00"[Date - Publication] AND "humans"[MeSH Terms] AND "english"[Language]))) AND ((y_5[Filter]) AND (humans[Filter]) AND (english[Filter])) | 24 |
| 4 | (tutorial OR framework OR method* or methodology(MeSH Terms) | ("tutorial"[All Fields] OR "tutorials"[All Fields] OR "framework"[All Fields] OR "framework s"[All Fields] OR "frameworks"[All Fields] OR "method*"[All Fields]) AND ((y_5[Filter]) AND (humans[Filter]) AND (english[Filter])) | 2,053,568 |
| 3 | (spatial OR GIS OR "spatial overlay" OR geocoding) | ("spatial"[All Fields] OR "spatialization"[All Fields] OR "spatializations"[All Fields] OR "spatialized"[All Fields] OR "spatially"[All Fields] OR ("proc acm sigspatial int conf adv inf"[Journal] OR "gis"[All Fields]) OR "spatial overlay"[All Fields] OR ("geocode"[All Fields] OR "geocoded"[All Fields] OR "geocoder"[All Fields] OR "geocodes"[All Fields] OR "geographic mapping"[MeSH Terms] OR ("geographic"[All Fields] AND "mapping"[All Fields]) OR "geographic mapping"[All Fields] OR "geocoding"[All Fields])) AND ((y_5[Filter]) AND (humans[Filter]) AND (english[Filter])) | 56,705 |
| 2 | (health OR public health) | ("health"[MeSH Terms] OR "health"[All Fields] OR "health s"[All Fields] OR "healthful"[All Fields] OR "healthfulness"[All Fields] OR "healths"[All Fields] OR ("public health"[MeSH Terms] OR ("public"[All Fields] AND "health"[All Fields]) OR "public health"[All Fields])) AND ((y_5[Filter]) AND (humans[Filter]) AND (english[Filter])) | 2,379,072 |
| 1 | ("web scraping" OR "web-mined" OR "crowdsourced") | ("web scraping"[All Fields] OR "web-mined"[All Fields] OR "crowdsourced"[All Fields]) AND ((y_5[Filter]) AND (humans[Filter]) AND (english[Filter])) | 555 |
