# Supplementary material for: A Multisource, Multilevel Contextual Database to Support Ending the HIV Epidemic Initiative: Protocol for Design, Construction, Harmonization, and Quality Assurance
Source: JMIR Res Protoc. 2026 Nov 3;15:e100648. doi: 10.2196/100648 (PMC13730519; doi:10.2196/100648)
Supplement: Supplementary file 2 [file resprot-v15-e100648-s002.docx]

**Multimedia Appendix 2**

Screening Criteria for Literature Search

**Database Methodology Study Eligibility**

**Screening Form**

Instructions: Screen based on title and abstract only. When uncertain, select “Maybe” and add a brief note.

**1.** Is the study published in English?

- ☐ Yes → Continue
- ☐ Unsure → Tag “Maybe” and add a note
- ☐ No → Exclude and tag “Wrong Language”

2. Is the study published between 2020 and 2025 (inclusive)?

- ☐ Yes → Continue
- ☐ Unsure → Tag “Maybe” and add a note
- ☐ No → Exclude and tag “Outside Time Window”

3. Does the study involve observational data derived from human subjects?

*(Includes routinely collected data such as EHRs, registries, surveillance systems, administrative data, claims, or linked population datasets.)*

- ☐ Yes → Continue
- ☐ Unsure → Tag “Maybe” and add a note
- ☐ No → Exclude and tag “Wrong Population”

4. Does the study involve quantitative data?

- ☐ Yes → Continue
- ☐ Unsure → Tag “Maybe” and add a note
- ☐ No → Exclude and tag “Wrong Study Type”

5. Does the study describe methods, frameworks, or protocols related to data management or database construction?

*(e.g., data collection workflows, harmonization, integration, quality assurance, governance, metadata, pipelines, or database architecture)*

- ☐ Yes → Include for full-text review
- ☐ Unsure → Tag “Maybe” and add a note
- ☐ No → Exclude and tag “Wrong Topic”

**Full-Text Eligibility Criteria**

**Include if the full text describes one or more of the following:**

- Data management or database methodology
- Data harmonization or linkage across sources
- Data quality assurance or validation procedures
- Data governance, provenance, or metadata standards
- Web scraping or automated data ingestion methods
- Construction, maintenance, or updating of observational databases
- Reproducible data pipelines or infrastructure for observational research
- Methodological frameworks applicable beyond a single analytic result

**Exclude at full text if the study is primarily:**

- A results-focused report using an existing database without substantive discussion of data methods
- A planned or active registry whose primary purpose is to support a clinical trial, *without* methodological contribution to data management
- A clinical trial protocol where database methods are ancillary rather than a central focus
- A descriptive epidemiologic or outcomes study with no novel or transferable data-methods component

**Tag exclusions as:**

- “Clinical Trial–Focused Registry”
- “Results Only / No Data Methods”
- “Not Methodological”

**Title & Abstract Screening**

**Database Proxy Variable Search Study Eligibility Screening Form**

Instructions: Screen based on title and abstract only. If eligibility is unclear, select “Maybe” and add a brief note.

1. Is the study published in English?

- ☐ Yes → Continue
- ☐ Unsure → Tag “Maybe” and add a note
- ☐ No → Exclude and tag “Wrong Language”

2. Is the study published between 2020 and 2025 (inclusive)?

- ☐ Yes → Continue
- ☐ Unsure → Tag “Maybe” and add a note
- ☐ No → Exclude and tag “Outside Time Window”

3. Does the study involve quantitative data?

- ☐ Yes → Continue
- ☐ Unsure → Tag “Maybe” and add a note
- ☐ No → Exclude and tag “Wrong Study Type”

4. Does the study describe the use of secondary data?

*(e.g., administrative data, EHRs, surveillance data, census or ACS data, geospatial indicators, registries, or other routinely collected datasets)*

- ☐ Yes → Include for full-text review
- ☐ Unsure → Tag “Maybe” and add a note
- ☐ No → Exclude and tag “No Data Use”

**Full-Text Eligibility Criteria**

**Include if the full text demonstrates that the study:**

- Uses quantitative contextual, area-level, or social determinants of health (SDOH) variables
- Incorporates these variables as proxies or inputs in the estimation, modeling, or interpretation of health outcomes
- Draws on secondary data sources (e.g., census, administrative records, policy databases, environmental or neighborhood measures) rather than exclusively primary survey instruments
- Clearly specifies how proxy variables are operationalized, linked, or applied within analytic models

**Exclude at full text if the study is primarily:**

- Non-quantitative (qualitative-only or narrative descriptions without analytic use of proxy variables)
- Duplicative, such as secondary publications using the same proxy measures without novel application or justification
- Reliant exclusively on internal, study-specific measures (e.g., bespoke survey items, trial-specific indices) with no externally derived contextual or SDOH data
- Descriptive discussions of SDOH or context without empirical use in outcome estimation

**Tag exclusions as:**

- “Non-Quantitative”
- “Duplicative”
- “Internal Measures Only”
- “No Proxy Variable Use”
