# Supplementary material for: A Multisource, Multilevel Contextual Database to Support Ending the HIV Epidemic Initiative: Protocol for Design, Construction, Harmonization, and Quality Assurance
Source: JMIR Res Protoc. 2026 Nov 3;15:e100648. doi: 10.2196/100648 (PMC13730519; doi:10.2196/100648)
Supplement: Supplementary file 3 [file resprot-v15-e100648-s003.docx]

**Multimedia Appendix 3**

Implementation of FAIR Data Principles

This appendix summarizes how the database development aligns with FAIR (Findable, Accessible, Interoperable, and Reusable) data principles. The goal of incorporating FAIR principles into this research is to facilitate transparency, reproducibility, and reusability of the data.

| ***FAIR Principle*** | ***Description*** | ***Implementation in Project*** |
| --- | --- | --- |
| **F**indable | Both data and metadata should be easy to find, assigned a unique identifier, and are registered or indexed in a searchable resource. | All data sources will be documented in a structured inventory with standardized naming conventions, version identifiers, and access links. Each dataset will be assigned an identifier to support citations and reproducibility. |
| **A**ccessible | Data and metadata are retrievable by their identifier using a standardized protocol which is open, free, and universally implementable. | Data and metadata will be retrievable through open and widely used protocols. The database protocol and all code used for development will be shared via a public GitHub repository upon project completion. Access conditions for each data source will be explicitly documented, and metadata will remain accessible even when data are restricted. |
| **I**nteroperable | Data and metadata are accessible and use vocabulary that followed FAIR principles, allowing for integration with other data. | Data will be harmonized using standardized geographic identifiers and consistent variable definitions. Common data structures and coding schemes are applied across sources to facilitate linkage. |
| **R**eusable | Data and metadata should be well-described, with clear and accessible data usage licenses, and documented with access links and other provenance. | Detailed documentation will be provided for data provenance, transformations, harmonization decisions, and quality checks. Version control and change logs support reproducibility. Citations and access links support reuse. |
