# Supplementary material for: A Multisource, Multilevel Contextual Database to Support Ending the HIV Epidemic Initiative: Protocol for Design, Construction, Harmonization, and Quality Assurance
Source: JMIR Res Protoc. 2026 Nov 3;15:e100648. doi: 10.2196/100648 (PMC13730519; doi:10.2196/100648)
Supplement: Supplementary file 4 [file resprot-v15-e100648-s004.docx]

**Multimedia Appendix 4**

**Figure S1.** *PRISMA 2020 Flow Diagram for Methods Literature Search^[1]^*

**Identification of studies via databases**

Records removed *before screening*:

Duplicate records removed

(n = 0)

Records identified from:

PubMed (n =80)

**Identification**

Records screened:

(n = 80)

Records excluded by human

(n = 64)

Reports sought for retrieval:

(n = 16)

Reports not retrieved due to restricted library access:

(n = 0)

**Screening**

Reports excluded:

Multicenter clinical or hospital-based protocols (n=3)

Registry development reports (n=2)

Studies using only single health system data (n=2)

Automated machine learning demonstrations (n=2)

Reports assessed for eligibility:

(n = 16)

Studies included in review:

(n = 7)

**Included**

**Figure S2.** *PRISMA 2020 Flow Diagram for Proxy Variable Literature Search^[2]^*

**Identification of studies via databases**

Records removed *before screening*:

Duplicate records removed

(n = 1)

Records identified from:

PubMed (n =274)

**Identification**

Records screened:

(n = 273)

Records excluded by human

(n = 210)

Reports sought for retrieval:

(n = 65)

Reports not retrieved due to restricted library access:

(n = 2)

**Screening**

Reports excluded:

Duplicate (n = 2)

No database mentioned (n =11)

Qualitative data only (n =12)

Reports assessed for eligibility:

(n = 63)

Studies included in review:

(n = 38)

**Included**

1. Adapted from *The PRISMA 2020 statement: An updated guideline for reporting systematic reviews* (Page et al., 2021).
2. Adapted from *The PRISMA 2020 statement: An updated guideline for reporting systematic reviews* (Page et al., 2021).
