# Supplementary material for: A Multisource, Multilevel Contextual Database to Support Ending the HIV Epidemic Initiative: Protocol for Design, Construction, Harmonization, and Quality Assurance
Source: JMIR Res Protoc. 2026 Nov 3;15:e100648. doi: 10.2196/100648 (PMC13730519; doi:10.2196/100648)
Supplement: Supplementary file 5 [file resprot-v15-e100648-s005.docx]

**Multimedia Appendix 5**

Data Sources Included in the Database to Date*

| ***Data Source*** | ***Geographic Level*** | ***Description*** | ***Years Included*** | ***Number of Datasets*** |
| --- | --- | --- | --- | --- |
| AIDSVu | County, State | HIV surveillance, healthcare continuum, and prevention data stratified for individuals with confirmed and diagnosed HIV aged 13 and older, mapped alongside community-level socio-environmental data. | 2020-2024 | County: *n* = 19  State: *n* = 24 |
| American Community Survey (ACS) 1-Year Estimates | County, State | Annual data reports from the U.S. Census Bureau that combines 12 months of community survey responses for social, economic, housing, and demographic data. | 2021-2024 | County: *n* = 44  State: *n* = 44 |
| America's HIV Epidemic Analysis Dashboard (AHEAD) | County, State | Data on the six Ending the HIV Epidemic (EHE) indicators for EHE priority jurisdictions as well as all states alongside social determinants of health (SDOH) data. | 2020-2025 | County: *n* = 1  State: *n* = 1 |
| Bureau of Justice Statistics | State | Criminal justice statistics that measure crime, criminal offenders, victims of crime, and the operation of justice systems at the federal, state, tribal, and local levels. | 2020-2023 | State: *n* = 3 |
| CDC AtlasPlus | County, State | An interactive tool that provides customized tables, maps, and charts of CDC surveillance data on HIV, viral hepatitis, STDs, and TB alongside SDOH indicators. | 2020-2024 | County: *n* = 5  State: *n* = 19 |
| CDC PLACES | County | Chronic disease and other health-related data for all U.S. counties, incorporated and census designated places, census tracts, and ZIP Code Tabulation Areas (ZCTAs) across the U.S. | 2020-2025 | County: *n* = 5 |
| CDC Social Vulnerability Index (SVI) | County | A place-based index, database, and mapping system that uses 16 U.S. Census variables from the 5-years ACS to identify communities in need before, during, or after disasters. | 2020-2022 | County: *n* = 42 |
| Centers for Medicare & Medicaid Services (CMS) | County, State | CMS health coverage, payment, and provider data alongside geographic coverage and quality ratings. | 2020-2025 | County: *n* = 6  State: *n* = 10 |
| County Health Rankings & Roadmaps | County | Annual data measuring vital health factors and outcomes including population health and well-being, physical environmental, social and economic factors, demographics, and other community conditions for nearly every county across all 50 U.S. states. | 2020-2025 | County: *n* = 6 |
| Health Resources & Service Administration (HRSA) | County, State | Maps, data, and dashboards on the U.S. healthcare workforce, federal grants, health center performance, and medically underserved areas. | 2020-2024 | County: *n* = 2  State: *n* = 2 |
| KFF | State | State-level health policy indicators including HIV/AIDS, health coverage, health insurance, Medicaid and CHIP, women’s health, health status, mental health, health disparities, health costs, and providers and service use data. | 2022-2025 | State: *n* = 47 |

**Note:* These data sources do not represent the final list of sources that are to be included in the database.
